# Deep6mA: A deep learning framework for exploring similar patterns in DNA N6-methyladenine sites across different species

**DOI:** 10.1371/journal.pcbi.1008767

**Published:** 2021-02-18

**Authors:** Zutan Li, Hangjin Jiang, Lingpeng Kong, Yuanyuan Chen, Kun Lang, Xiaodan Fan, Liangyun Zhang, Cong Pian

**Affiliations:** 1 Department of Mathematics, College of Science, Nanjing Agricultural University, Nanjing, China; 2 Center for Data Science, Zhejiang University, Hangzhou, China; 3 College of information science & Technology, Nanjing Agricultural University, Nanjing, China; 4 Department of Statistics, The Chinese University of Hong Kong, Hong Kong SAR, China; DAL, CANADA

## Abstract

N6-methyladenine (6mA) is an important DNA modification form associated with a wide range of biological processes. Identifying accurately 6mA sites on a genomic scale is crucial for under-standing of 6mA’s biological functions. However, the existing experimental techniques for detecting 6mA sites are cost-ineffective, which implies the great need of developing new computational methods for this problem. In this paper, we developed, without requiring any prior knowledge of 6mA and manually crafted sequence features, a deep learning framework named Deep6mA to identify DNA 6mA sites, and its performance is superior to other DNA 6mA prediction tools. Specifically, the 5-fold cross-validation on a benchmark dataset of rice gives the sensitivity and specificity of Deep6mA as 92.96% and 95.06%, respectively, and the overall prediction accuracy is 94%. Importantly, we find that the sequences with 6mA sites share similar patterns across different species. The model trained with rice data predicts well the 6mA sites of other three species: *Arabidopsis thaliana*, *Fragaria vesca* and *Rosa chinensis* with a prediction accuracy over 90%. In addition, we find that (1) 6mA tends to occur at GAGG motifs, which means the sequence near the 6mA site may be conservative; (2) 6mA is enriched in the TATA box of the promoter, which may be the main source of its regulating downstream gene expression.

## Author summary

DNA N6 methyladenine (6mA) is a newly recognized methylation modification in eukaryotes. It exists widely and conservatively in organisms, and its modification level changes dynamically in the whole life cycle. This study proposes an algorithm based on a deep learning framework including LSTM and CNN to predict 6mA sites. The results showed that our method could accurately predict the 6mA sites in different species, which means DNA sub-sequences containing 6mA sites among species have certain conservation. Importantly, we found that 6mA methylation in most different species is more likely to occur on the GAGG motif. In addition, we also found that 6mA is rich in the promoter’s TATA box, which may be a mechanism of regulating downstream gene expression.

This is a *PLOS Computational Biology* Methods paper.

## Introduction

DNA methylation modifications such as N4-methylcytosine (4mC), N6-methyladenine (6mA), and 5-methylcytosine (5mC) play important roles in epigenetic regulation of gene expression without altering the sequence, and it is widely distributed in the genome of different species [1]. DNA N6-methyladenine (6mA) refers to the methylation of the 6^th^ nitrogen atom of adenine, which has been found to play an important role in the epigenetic modification of eukaryotic DNA in recent years [2]. Previous studies have shown that 6mA plays important roles in DNA repair [3–4], DNA replication [5], regulating gene transcription [6] and gene expression regulation [7]. Although 6mA sites are not uniformly distributed across the genome and they may be affected by environmental factors [8], the methylation protection is a genetic state, and 6mA in prokaryotes and eukaryotes shows similar characteristics [9]. DNA 6mA on the genome is essential to reveal the detail of epigenetic modification process.

Due to recent advances in high-throughput sequencing technologies, various experimental techniques were reported to promote the study of 6mA distribution and its potential function in genome of eukaryotes and prokaryotes. For example, Pormraning et al. [10] applied sequencing of methylated DNA immunoprecipitation technique to reveal the presence of DNA methylation in eukaryotes. Krais et al. [11] reported that the capillary electrophoresis with laser-induced fluorescence can be used to detect global adenine methylation in DNA. Meanwhile, Flusberg et al. [12] used a method based on single-molecule, real-time sequencing technique to detect DNA methyladenine directly. Greer et al. [13] developed a method with the ultra-high-performance liquid chromatography and the mass spectrometry to discover the signals of DNA 6mA sites. These methods advanced the research of 6mA. By using 6mA-IP-Seq, Fu et al. [14] found that 84% of 6mA modification exit in Chlamydomonas genes. Koziol et al. [15] identified the 6mA modification in vertebrates by using HPLC, blots, and sequencing of methylated DNA Immunoprecipitation (MeDIP-seq). Zhou et al. [16] found through 6mA immunoprecipitation, mass spectrometry, and single molecule realtime that 0.2% of adenines in the rice genome are 6mA methylated and GAGG-rich sequences are the most significantly enriched for 6mA.

To date, tools were developed to predict 6mA methylation modification. For instance, Chen et al. [17] proposed a method called i6mA-Pred to identify DNA 6mA sites based on the support vector (SVM) with 164 chemical features of nucleotides and position-specific nucleotide frequencies. The i6mA-Pred shows a good classification performance in rice 6mA data. However, it does not fully capture the information between nearby nucleotides. To address this weakness, Pian et al. [18] used a first-order Markov model, MM-6mAPred, to predict 6mA sites. Their results show that the significant difference of the transfer probabilities of adjacent nucleotides is the key to the improved performance of MM-6mAPred. Kong et al. [19] employed the Dinucleotide composition and dinucleotide-based DNA properties to represent DNA sequences, it also used a bagging classifier to build the prediction model which called i6mA-DNCP. Compared with i6mA-Pred and i6mA-DNCP, MM-6mAPred has a better performance in terms of prediction accuracy. Shaherin et al. [20] developed a predictor called SDM6A which explored various features and five encoding methods for identifying DNA 6mA sites. Liu et al. [21] proposed a machine learning-based prediction tool named csDMA which used three feature encoding schemes, Motif, Kmer and Binary to generate the feature matrix.

The above five prediction models were trained on the 880 rice 6mA sites and 880 non-6mA sites [17]. They did not consider the complex structure information in the sequence such as linkage disequilibrium between nucleotides, thus, there is still some room to improve. Zhou et al. [16] found 265,290 rice 6mA sites through a variety of experimental methods, such as HPLC-MS/MS, 6mA immunoprecipitation sequencing and Single Molecule Real-Time (SMRT), which enables us to train complex models for 6mA identification. For example, Lv et al. [22] provided a new random forest model named iDNA6mA-rice based on the reconstructed 154,000 6mA sites and 154,000 non-6mA sites. iDNA6mA-rice is mainly realized by the random forest algorithm module (RF) based on three feature extraction techniques: K-tuple nucleotide frequency component, mono-nucleotide binary encoding and natural vector. Based on Convolutional Neural Networks (CNN), Yu and Dai [23] proposed a method named SNNRice6mA to predict the 6mA sites of rice, and showed its advantages over other methods. It is known to us that there is a strong dependence between nucleotides on the sequence, especially on the conserved DNA sequences, thus, the key difficulty in 6mA prediction is to take into consideration this dependence structure in statistical modeling. However, as we known, CNN is limited in learning information about long-distance dependence although it has a strong learning ability in the fields of image recognition, voice recognition and agricultural intelligence [24–27].

Recurrent Neural Network (RNN) is a special neural network structure, inspired by the fact that human cognition is based on past experience and memory. Different from CNN, RNN not only considers the input of the previous moment, but also can effectively "remember" the previous content. Therefore, RNN has an advantage in analyzing the sequence containing timing information. At present, RNN has been widely used in fields such as natural language processing, image processing, machine translation, speech recognition and bioinformatics [28–30]. However, it is difficult to train an RNN due to gradient disappearance or gradient explosion. Long Short-Term Memory (LSTM) and Gate Recurrent Unit (GRU) are proposed to overcome this difficulty, and they are the most commonly used RNNs.

In this study, we introduced a novel deep learning framework named Deep6mA to identify DNA 6mA sites. Deep6mA composed of a CNN and a bidirectional LSTM (BLSTM) module is shown to have a better performance than other methods on 6mA prediction. Interestingly, we find that the motif with the highest frequency of 6mA methylation is concentrated on GAGG among four plant species: *Rice*, *Arabidopsis thaliana*, *Fragaria vesca* and *Rosa chinensis*, which means the 6mA methylation has similar patterns across different species. This is further evidenced by the fact that the model trained by rice data has a high accuracy to predict 6mA in other three species. We may conclude from these results that the sequence prone to DNA 6mA methylation among different species is conservative, and Deep6mA may also be applicable to analyze the 6mA site of other plant species. More importantly, we found that 6mA is generally enriched in TATA box of the promoter. This may be an important way for 6mA to regulate gene expression.

## Results

### Comparing CNN with CNN+LSTM

In this section, we compare the performance of CNN with CNN + LSTM based on the same training data under different settings of CNN. Note that we use the same structure of CNN to compare these two methods. The number of convolution layer in CNN and CNN+LSTM models is set as 1, 2, or 3, the corresponding convolution kernel size is set as 5, 8, 10, or 16 (discussion on selecting the number of CNN layers and kernel size is given in S2 Fig) and the number of convolution kernel is set as 256. Finally, the unit number of LSTM in CNN+LSTM models is set to 32. Table 1 shows the performance of these two methods under different convolution layers and kernel sizes. The result shows that the performance of CNN + LSTM is better than that of CNN, due to the ability of LSTM to learn the dependence structure underlying the sequence.

**Table 1:** The performance of CNN and CNN + LSTM based on 6mA-rice-Lv dataset under different CNN layers and kernel sizes.

	Model	CNN layers	Kernel size	SP (%)	SN (%)	ACC (%)	MCC	AUC
CNN	1-256-5	1	5	65.37	72.81	69.09	0.38	0.76
	1-256-8	1	8	84.00	65.56	74.78	0.51	0.84
	1-256-10	1	10	83.73	69.34	76.53	0.54	0.86
	1-256-16	1	16	79.95	86.85	83.40	0.67	0.91
	2-256-5	2	5	80.94	83.87	82.41	0.65	0.90
	2-256-8	2	8	70.33	94.13	82.23	0.67	0.92
	2-256-10	2	10	80.21	96.97	88.59	0.78	0.97
	2-256-16	2	16	91.98	94.53	93.26	0.87	0.98
	3-256-5	3	5	66.39	93.94	80.17	0.63	0.91
	3-256-8	3	8	82.14	96.89	89.52	0.80	0.97
	3-256-10	3	10	86.19	97.09	91.64	0.84	0.97
	3-256-16	3	16	86.88	96.85	91.86	0.84	0.98
CNN +LSTM	1-256-5-32	1	5	71.86	88.13	80.00	0.61	0.88
	1-256-8-32	1	8	89.82	95.02	92.42	0.85	0.97
	1-256-10-32	1	10	91.39	95.33	93.36	0.87	0.98
	1-256-16-32	1	16	91.91	94.93	93.42	0.87	0.98
	2-256-5-32	2	5	86.73	91.80	89.26	0.79	0.95
	2-256-8-32	2	8	92.63	94.67	93.65	0.87	0.98
	2-256-10-32	2	10	92.57	94.70	93.63	0.87	0.98
	2-256-16-32	2	16	92.19	95.11	93.65	0.87	0.98
	3-256-5-32	3	5	88.67	92.76	90.72	0.82	0.96
	3-256-8-32	3	8	92.49	93.56	93.03	0.86	0.97
	3-256-10-32	3	10	93.33	94.03	93.68	0.87	0.98
	3-256-16-32	3	16	93.09	94.55	93.82	0.88	0.98

In addition, we use 6mA-rice-Chen and Fragaria vesca data as additional independent verification datasets to test whether this marginal improvement of CNN + LSTM is applicable to other data. The results from S1 and S2 Tables show that the performance of CNN + LSTM is better than that of CNN when they have the same CNN structure.

### Selecting model parameters of CNN+LSTM

**Fig 1:**
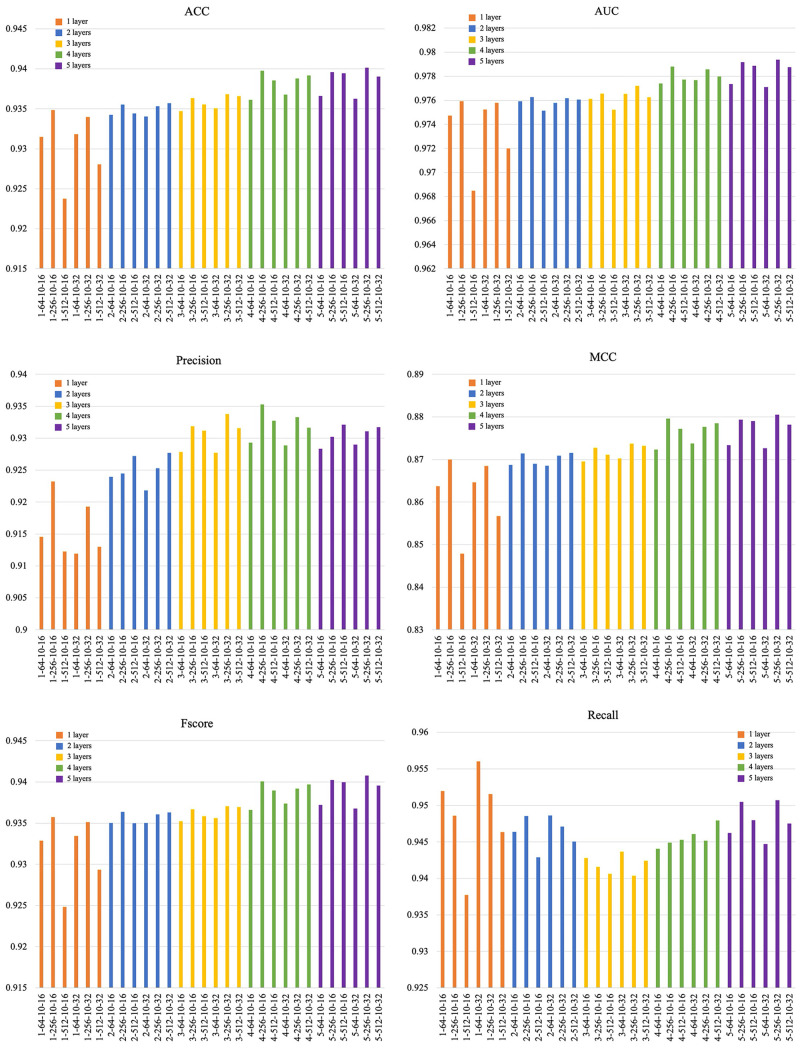
Prediction performance of CNN+LSTM model under different settings of parameters. The CNN+LSTM with parameter 5-256-10-32 is chosen as our final CNN+LSTM model (convolution layer: 5, convolution kernel: 256, filter size: 10, hidden units of LSTM: 32).

It is known that the performance of CNN+LSTM framework depends on the filter size and the number of convolution kernels of the convolution layer, and number of hidden units in LSTM. To simplify notations, we denote the CNN+LSTM framework with x convolution layer(s), y convolution kernel(s), z filter size and w hidden units as a CNN+LSTM with parameter x-y-z-w. In this section, we select the best CNN+LSTM model from 30 different settings of parameter x, y, z, w by 5-fold cross-validation. Specifically, we take x from {1, 2, 3, 4, 5}, y from {64, 256, 512}, w from {16, 32} and fix z at 10. Fig 1 shows the prediction performance of CNN+LSTM model under different settings of parameters. According to these results, the CNN+LSTM with parameter 5-256-10-32 is chosen as our final CNN+LSTM model.

### Location features of 6mA sites

**Fig 2:**
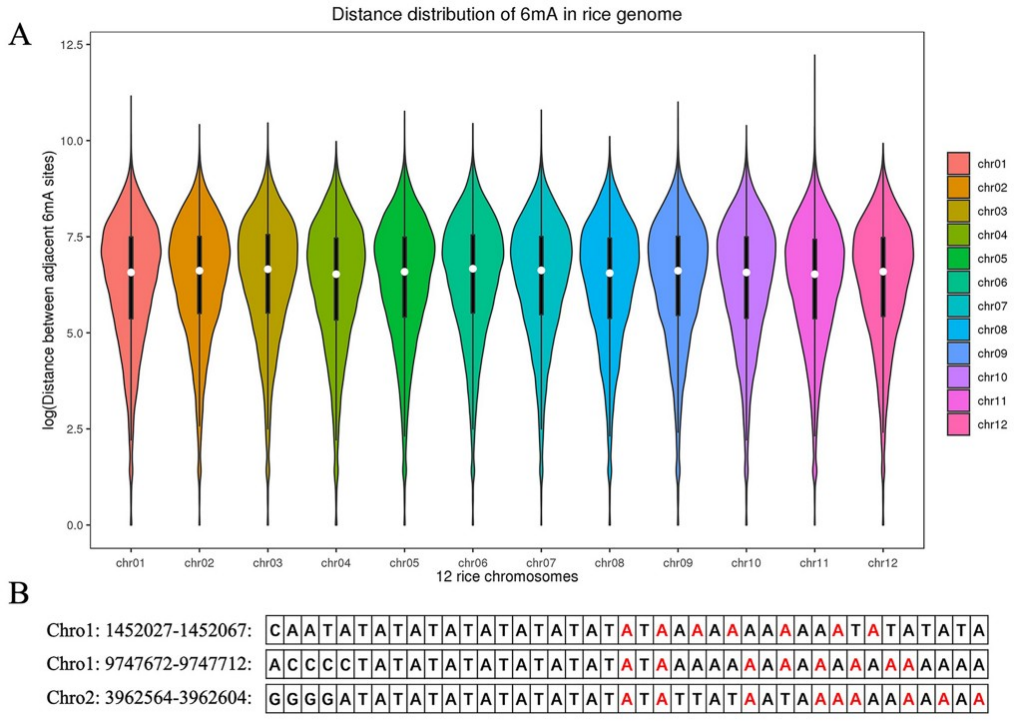
Location features of 6mA sites in the sequence. (A) The distribution of 6mA methylation modification on 12 chromosomes. The X axis and Y axis represent the 12 chromosomes and the logarithm of distance between adjacent 6mA site. (B) Three examples of sequences containing TATA box enriched with 6mA methylation (colored in red).

In this section, we investigate the location features of 6mA sites, that is, to see whether 6mA methylation is enriched in a contiguous region of the genome. Fig 2A shows the distribution of the distance between adjacent 6mA sites in 12 chromosomes. According to the results, we find that (1) the distributions of the distance between adjacent 6mA sites are similar for different chromosomes; and (2) the mean of the distance between adjacent 6mA sites is greater than 64nt, which indicates that 6mA sites seldom occur in a continuous region like 5mC sites to form a DMR. To further investigate the location feature of 6mA sites which indeed cluster together, we look inside the subsequences of length 30nt with more than five 6mA sites, and find that almost all of the 6mA sites in such subsequences are located in the TATA boxes of the promoters (see Fig 2B for examples, more details are given in S3 Table). This implies that 6mA may, in general, be enriched in TATA box, which is an important functional component of the promoter. The transcription process will not start until RNA polymerase binds tightly on TATA box. Therefore, the enrichment of 6mA methylation on TATA box may directly affect the expression of downstream genes. This may be an important regulatory function of 6mA methylation modification.

### Comparison with other leading methods

It is shown that classification performance of MM-6mAPred is better than those of i6mA-Pred, iDNA6mA, csDMA, SDM6A and i6mA-DNCP based on the same data set (880 positive samples+880 negative samples) [17,19–22], and the performance of SNNRice6mA is better than that of iDNA6mA-Rice [22–23]. Therefore, we only compare Deep6mA with MM-6mAPred and SNN-Rice6mA. We use 5-fold cross-validation to evaluate the performance of Deep6mA, SNNRice6mA and MM-6mAPred based on 6mA-rice-Lv dataset (see Section “Benchmark dataset”). For SNNRice6mA, the number of convolution layer and convolution kernel is set to 1 and 4, respectively, and the filter size and fully connect layer size of SNNRice6mA is set to 3 and 64, respectively. Deep6mA is the best one among these methods in terms of SN, SP, ACC, MCC and AUC as shown in Table 2 with SN, SP, ACC, MCC and AUC as 95.06%, 92.96%, 94.01%, 0.88 and 0.98 respectively. The better performance of Deep6mA is mainly due to the ability of BLSTM to learn the dependence structure between distant nucleotides. To be specific, BLSTM is able to get useful information from a previous position for current position, and the distance between these two positions is adaptive to the sequence, maybe as short as 1 bp, or as long as 100 bp. This exactly matches the feature of 6mA sites that the distance between 6mA sites varies, since the position of the 6th nitrogen atom of adenine varies. In other words, BLSTM learns from training data how to predict the 6mA status of current site by using 6mA status of previous sites, and it also learns how many previous sites are used.

**Table 2:** Comparison of Deep6mA, SNNRice6mA and MM-6mAPred based on 6mA-rice-Lv dataset.

Method	SN (%)	SP (%)	ACC (%)	MCC	AUC
Deep6mA	**95.06**	**92.96**	**94.01**	**0.88**	**0.98**
SNNRice6mA-large	94.33	89.75	92.04	0.84	0.97
MM-6mAPred	93.47	89.51	91.49	0.83	0.96

**Fig 3:**
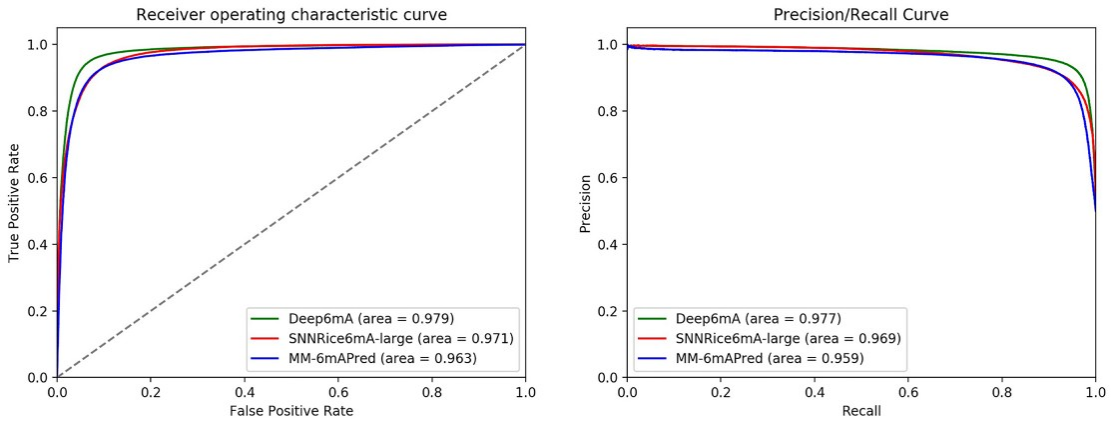
The ROC curves (A) and PRC curves (B) of Deep6mA based on 6mA-rice-Lv dataset.

In addition, the ROC curve and PR curve of Deep6mA, SNNRice6mA and MM-6mAPred are shown in Fig 3. The area under curve of Deep6mA is 0.979, which is higher than that from other two methods. All these results show that our method Deep6mA is the best one among these methods.

To further verify the performance of our proposed method, we also compared the prediction performance of Deep6mA, SNNRice6mA-large and MM-6mAPred based on the 6mA-rice-Chen dataset (880 positive and negative samples) [23]. The results shown in Table 3 indicate that the performance of Deep6mA is also better than those of SNNRice6mA-large and MM-6mAPred for the 6mA-rice-Chen dataset.

**Table 3:** Comparison of Deep6mA, SNNRice6mA-large and MM-6mAPred based on 6mA-rice-Chen dataset.

Method	SN (%)	SP (%)	ACC (%)	MCC	AUC
**Deep6mA**	**79.73**	**96.40**	**88.06**	**0.77**	**0.96**
**SNNRice6mA-large**	77.90	87.43	82.67	0.65	0.89
**MM-6mAPred**	76.82	91.70	84.26	0.68	0.91

### Validation on other three plant species

**Fig 4:**
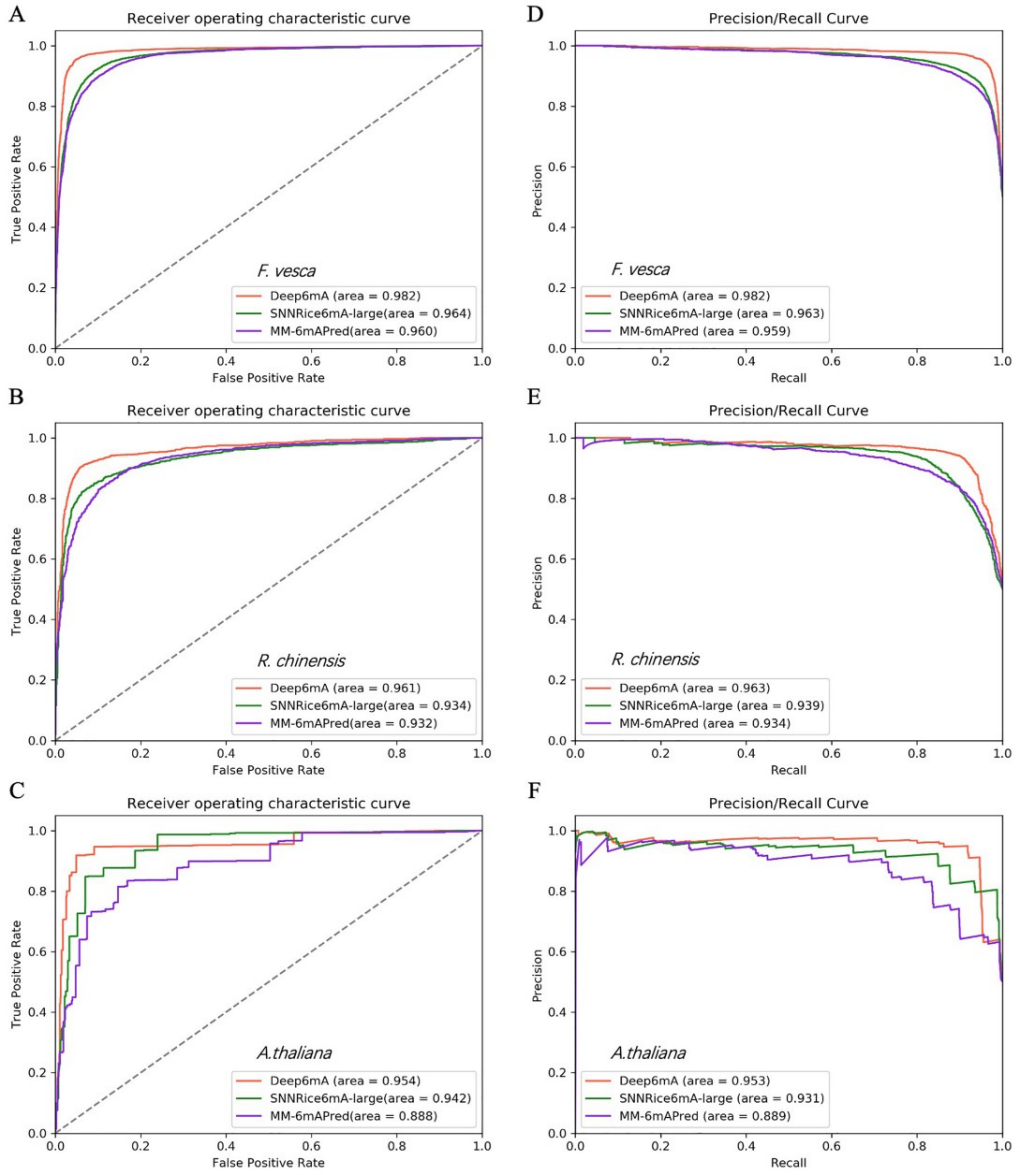
The ROC curves (A, B and C) and PRC curves (D, E and F) of three models (Deep6mA, SNNRice6mA-large and MM-6mAPred) based on three datasets (*F*.*vesca*, *R*.*chinensis* and *A*.*thaliana*).

Results in Section “Comparison of motifs across different species” show that 6mA is conservative among different species, which suggest that Deep6mA trained on rice data is applicable to predict the 6mA sites of other species. In the following, we try to validate this principle by applying the trained Deep6mA to the 6mA data of other plant species: *Arabidopsis thaliana* with sample size 98483, *Fragaria vesca* with positive and negative sample size 1417, and *Rosa chinensis* with sample size 5733 (see Section “Benchmark dataset” for details). i6mA-DNCP was not compared in this part because the performance of i6mA-DNCP is not as good as that of MM-6mAPred in rice 6mA data. The prediction results on these three test datasets are listed in Table 4. We found that Deep6mA trained with rice 6mA data predicts with high accuracy the 6mA sites in other three species. The prediction performance of our method is better than SNNRice6mA and MM-6mAPred. In addition, we also draw ROC and PRC curves (Fig 4), and the results show that the performance of our Deep6mA is better than the other two tools.

**Table 4:** Prediction accuracy of Deep6mA, SNNRice6mA and MM-6mAPred trained with rice data on *Fragaria vesca*, *Rosa chinensis* and *Arabidopsis thalian*a.

	*F*. *vesca*		*R*. *chinensis*		*A*.*thaliana*	
Model	SN	SP	SN	SP	SN	SP
**Deep6mA**	**0.94**	**0.95**	**0.87**	**0.95**	**0.98**	**0.96**
SNNRice6mA-large	0.92	0.84	0.84	0.85	0.91	0.92
MM-6mAPred	0.96	0.76	0.92	0.75	0.93	0.72

### Comparison of motifs across different species

**Fig 5:**
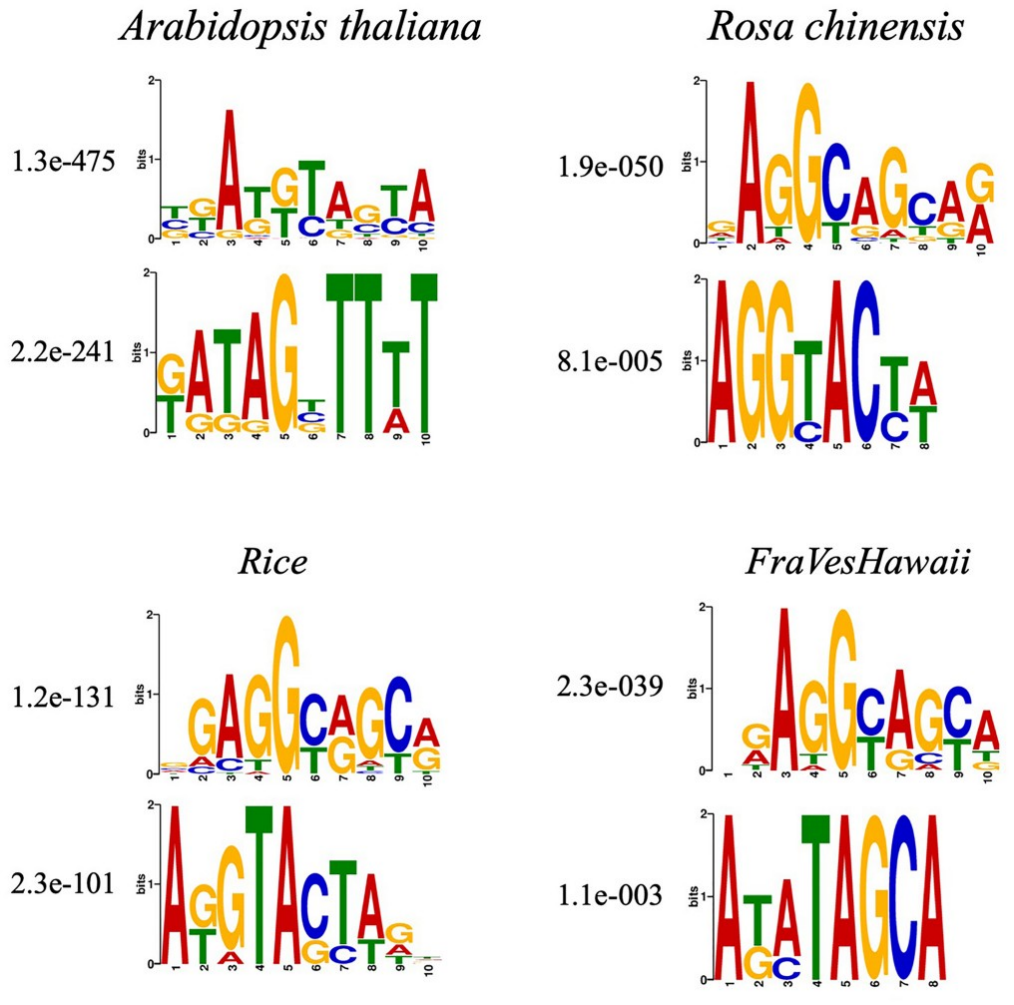
The top two significant motifs in the sequence near the 6mA site of four species. (A: *Arabidopsis thaliana*, B: *Rosa chinensis*, C: *Rice* and D: *Fragaria vesca*).

As shown in previous section, Deep6mA trained with rice data predict well the 6mA sites of the other three species, which implies that 6mA sequences of these different species should be conservative in some structure. This motivates us to compare motifs from these species. Firstly, MEME algorithm [31], a popular motif discovery method (available at http://meme-suite.org/tools/meme), is used for analyzing sequences with experimentally verified 6mA sites (*Rice*: 154,000, *Arabidopsis thaliana*: 98483, *Fragaria vesca*: 5733 and *Rosa chinensis*: 1417). Fig 5 shows the two most significant motifs for each specie. The result indicates that GAGG is the most significantly associated motif in these four species. Hence, we infer that DNA 6mA methylation occurs most frequently at GAGG motifs across different species. Xiao et al. [32] also pointed out that (G/C) AGG (C/T) is the most frequent motif in human genome. This suggests that the sequence near the DNA 6mA methylation site may be conserved among different species.

**Fig 6:**
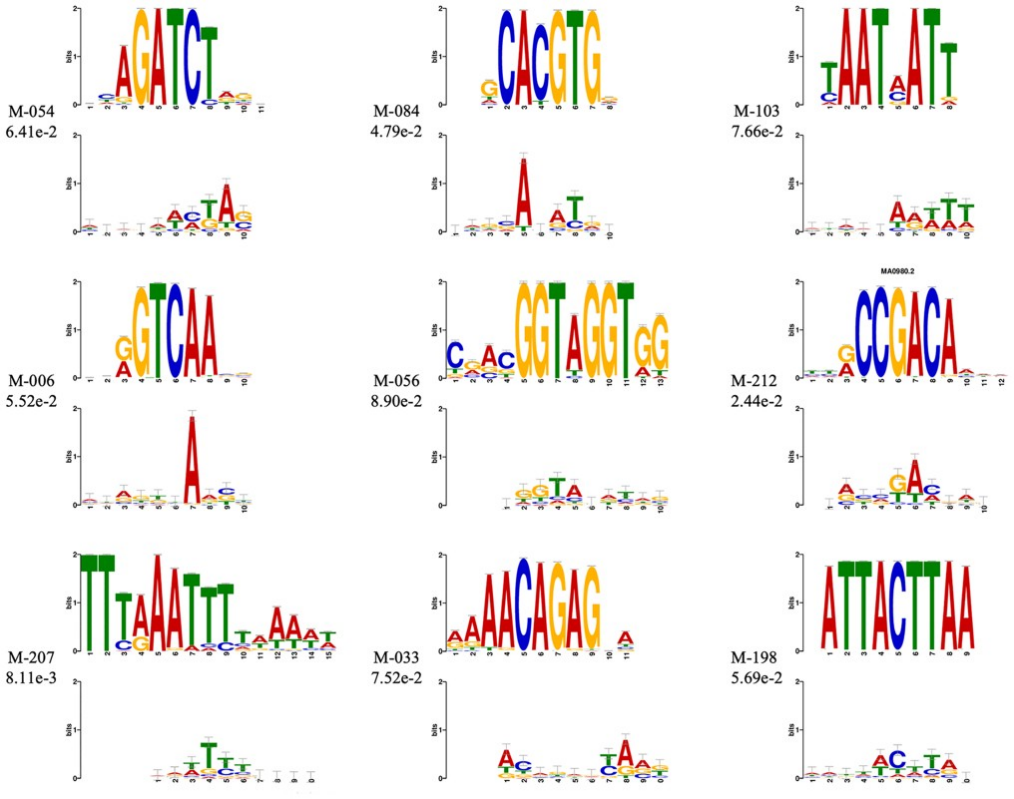
Significant motifs (E-value < 0.01) in *Rice*. The learned motifs from the first CNN layer of model are aligned with known motifs by means of TOMTOM.

We are not sure whether Deep6mA learns similar pattern from the training data (rice data), although Deep6mA is a deep learning framework which is able to learn DNA sequence patterns by discovering more new motifs [33]. To further understand the prediction ability of rice data trained Deep6mA on other species, we obtain 17 significant motifs learned from the first convolution layer of our model. Fig 6 shows the most significant 9 of them, and the remaining 8 are shown in S1 Fig. We can see from Fig 6 the AGG motif found by MEME is also in the list, which is conserved among different species.

## Discussion

In this study, we propose a deep learning framework named Deep6mA by integrating CNN and LSTM to efficiently predict DNA 6mA sites. Deep6mA uses a CNN layer to extract DNA sequence characterization, and then spreads it into a BLSTM layer to capture context dependency information of 6mA sites. Finally, these features are transferred to a fully connected layer to determine whether the site is a 6mA site. The experimental results show that Deep6mA can predict the 6mA site of rice with high accuracy. Importantly, we found that most of the 6mA methylation modifications in different plant species are more likely to occur on GAGG motifs. This shows that DNA sub-sequences containing 6mA sites among species have certain conservation. Maybe this is the reason why Deep6mA model trained with rice data can accurately predict 6mA sites in other plant species. However, there are some inadequacies in this study, such as the selection of sequence length. In theory, the longer the sequence, the more information it provides. All previous studies on 6mA site recognition are based on the sequence with a length of 41nt. It is not necessary to learn the complete sequence information by only training the model with those short sequences. Besides, due to the relative complexity of the calculation time, the framework and parameter design of Deep6mA may only achieve a local optimum. What’s more, why is the 6mA site enriched in the TATA box of the promoter, and whether this enrichment has a regulatory effect on the expression of downstream genes. For the ongoing work, we will carry out further research on these issues.

## Materials and methods

### Benchmark dataset

A benchmark dataset is important to build a reliable prediction model. In this study, for convenience, we use the 6mA-rice-Lv dataset [16,22], including 154,000 positive samples and 154,000 negative samples, to evaluate the proposed method and to compare it with other methods. For each positive sample obtained from GEO, the sequence is 41nt long with the 6mA site locating at the center. For each negative sample collected from NCBI, it’s also a sequence with length 41nt but contains no 6mA modification proved by experiments. In order to demonstrate that 6mA shares the similar patterns across different species and our method can also be used to detect DNA 6mA sites of other plant species, we also collected DNA 6mA sequences of *Arabidopsis thaliana*, *Fragaria vesca* and *Rosa chinensis* to show the ability of the trained Deep6mA from rice data on predicting 6mA methylation in these species. The 98483 6mA data of *Arabidopsis thaliana* is obtained from NCBI Gene Expression Omnibus (GEO) with accession number GSE81597. Negative samples for *Arabidopsis thaliana* were also collected. These samples are 41nt long sequences with Adenine in the center and were proved to be un-methylated by experiments. The i6mA-Fuse dataset [34], extracted from MDR [35] database, consisting of 5733 positive and negative samples for *R*. *chinensis*, and 1417 positive and negative samples for *F*. *vesca*.

### Sequence representation

Instead of using manually crafted DNA sequences features, we use the one-hot encoding method to convert the sequence into encoding tensor. Specifically, A, C, G, T, and N are encoded as (1,0,0,0), (0,1,0,0), (0,0,1,0), (0,0,0,1), and (0,0,0,0) respectively. Here the letter ‘N’ represents a non-sequenced nucleotide. Thus, the input DNA sequence is represented as a 4 by 41 encoding matrix, and is viewed as an image which motivates our design of deep learning framework.

### Convolutional neural network and long short-term memory network

Convolutional Neural Network (CNN) is widely used in image processing and speech recognition due to its high learning efficiency. The architecture of CNN is similar to that of the connectivity pattern of neurons in the human brain and was inspired by the organization of the visual cortex. A CNN generally consists of three parts: convolution layers, pooling layers and fully connected layers, which enables it to successfully capture the spatial and temporal dependence in an image. The convolution layer extracts the high-level features such as edges, color and gradient orientation through multiple feature mapping. The resolution of feature mapping is compressed further by a pooling layer to extract dominant features which are rotational and positional invariant, and to decrease the computational power required to process the data. After multiple convolution and pooling processes, the learned features are mapped to the sample label space by adding the full connection layer to achieve the purpose of classification and prediction.

Although CNN is powerful in image processing, it does not consider the dependence between inputs, and has a low power in sequence analysis such as natural language processing. Recurrent Neural Network (RNN) is proposed to overcome this shortcoming. As a special type of RNN, Long Short Term Memory Network (LSTM) is not only designed to capture the long dependent information in sequence but also overcome the training difficult of RNN due to gradient explosion and disappearance [36], thus it is the most widely used RNN in real applications. In LSTM, a storage mechanism is used to replace the hidden function in traditional RNN, with a purpose to enhance the learning ability of LSTM for long-distance dependency. Bi-directional LSTM (BLSTM), compared with unidirectional LSTM, captures better the information of sequence context.

### The Deep6mA model

**Fig 7:**
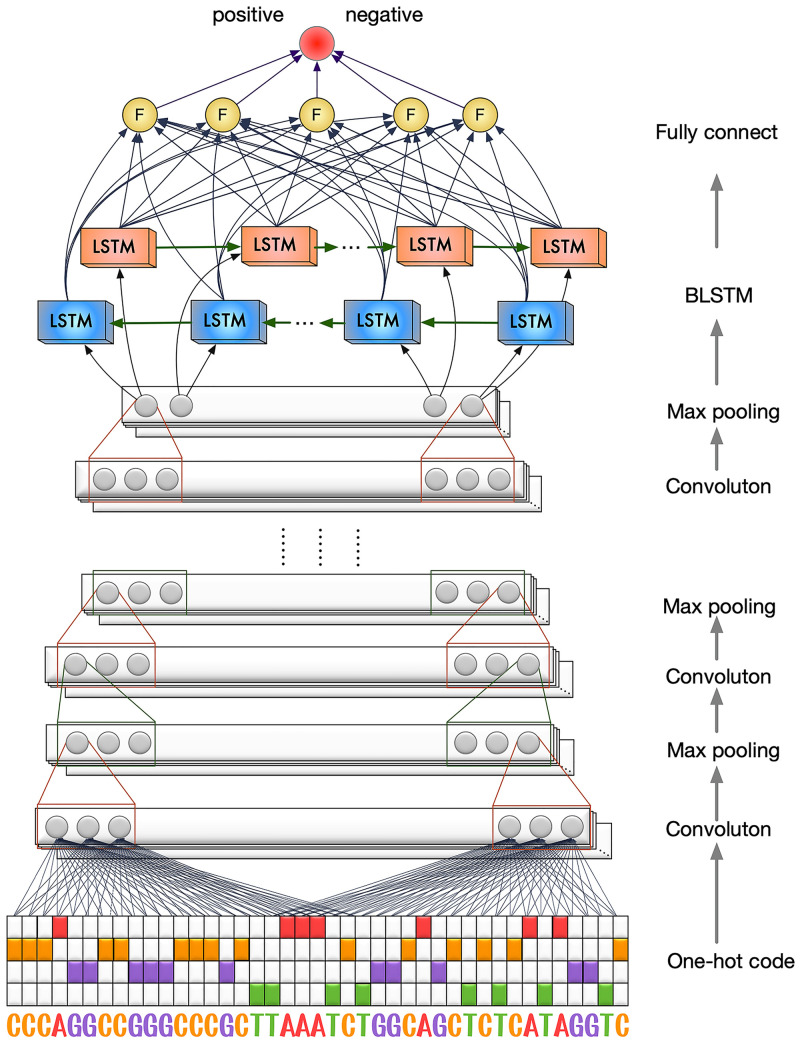
The flowchart of Deep6mA. The structure of Deep6mA consists of five layers of CNN, one layer of BLSTM and one layer of full connection layer.

The loci in DNA sequence are known to have a strong linkage disequilibrium, however, it is difficult to take into consider the dependence structure in traditional modeling for predicting 6mA sites. In this section, to fully capture the information in the sequence, we introduce a deep learning network, Deep6mA, which has a CNN to extract high-level features in the sequence and a BLSTM to learn dependence structure along the sequence. Specifically, Deep6mA is consist of five layers of CNN, one BLSTM layer and one fully connected layer. The convolution layer in CNN collocates 256 filters, and each filter size is 10. The exponential linear unit (ReLU) was used in CNN layers as activation function.

$$ReLU(x)=\{\begin{array}{ll} 0, & if\;x<0\\ x, & else \end{array}$$

where x is the feature map from the convolution operation. By viewing the input sequence as an image (see Section “Sequence representation”), the first convolution layer plays a role as motif detector of the 6mA sites in genome, while the other convolution layers capture higher-level features underlying the sequence. After each convolution layer, a pooling layer with Max Pooling is added to optimize the redundancy of the features and prevent overfitting. Then, one BLSTM layer with size 32 is added after CNN to learn the dependence structure in the sequence. The activation function used in this layer is the tanh activation function. In addition, a Fully Connected (FC) layer with 32 hidden units was used in this model. Finally, a sigmoid activation function is used to combine the outputs from the FC layer to make the final decision. Fig 7 shows the flowchart of Deep6mA.

The loss function for training Deep6mA is set as the binary cross-entropy measuring the difference between the target and the predicted output.

$$L(w)=-\sum_{i=1}^{N}y_{i}log\left(y_{i}^{\prime }\right)+(1-y_{i})log\left(1-y_{i}^{\prime }\right)+\alpha \|w{\|}_{2}$$

Where *y*_*i*_ is the true label, $y_{i}^{\prime }$ is the corresponding predicted value from Deep6mA, and *α*‖*w*‖_2_ is a regularization term to avoid overfitting.

Deep6mA is trained by using Adam [37]. Batch normalization and dropout [38] are applied after each convolutional procedure to accelerate training and avoid overfitting. The dropout rate is set as 0.5, the learning rate is set as 0.01, and the reduced factor is set as 0.5. In addition, the maximum training epoch and batch size is set as 50 and 256, respectively. We take 1/8 of training data, about 10% of the whole dataset, as validation data, and use an early stopping strategy with patience 5, which means the training process will stop when prediction performance did not improve on the validation set. The whole framework is implemented in Pytorch (https://pytorch.org).

### Prediction accuracy assessment

In this work, the prediction accuracy (ACC), Matthews correlation coefficient (MCC), sensitivity (SN) and specificity (SP) are used to evaluate the performance of different methods. Their definitions are given below. The receiver operating characteristic curve (ROC), the area under the curve (AUC) and precision recall curves (PRC) are used to show the detailed performance of different methods. The X-axis of the ROC curve is false positive rate (FPR = 1-SP), and the Y-axis is true positive rate (TPR = SN). The X-axis of the PRC curve is recall (Recall = SN), and the Y-axis is precision. The evaluation and comparison of the models in this paper are based on 5-fold cross validation.

$$S_{N}=\frac{T_{P}}{T_{P}+F_{N}}$$

$$S_{P}=\frac{T_{N}}{T_{N}+F_{P}}$$

$$Precision=\frac{T_{P}}{T_{P}+F_{P}}$$

$$ACC=\frac{T_{P}+T_{N}}{T_{P}+{T_{N}+F_{P}+F}_{N}}$$

$$MCC=\frac{T_{P}\times T_{N}-F_{P}\times F_{N}}{\sqrt{\left(T_{P}+F_{P})\times (T_{N}+F_{N})\times (T_{P}+F_{N})\times (T_{N}+F_{P}\right)}}$$

where TP is the number of real 6mA sequences predicted correctly as 6mA methylated, TN is the number of non-6mA sequences correctly predicted as non-6mA methylated, FN is the number of 6mA sequences predicted incorrectly as non-6mA methylated and FP is the number of non-6mA sequences predicted incorrectly as 6mA methylated.

## Supporting information

S1 Table The performance of CNN and CNN + LSTM based on 6mA-rice-Chen dataset under different CNN layers and kernel sizes. (DOCX)

S2 Table The performance of CNN and CNN + LSTM based on *F*.*vesca* dataset under different CNN layers and kernel sizes. (DOCX)

S3 Table Sequence containing 6mA site enriched in TATA box of promoter region in Rice. (XLS)

S1 Fig The remaining eight significant motifs from the first layer of CNN in Rice. (JPG)

S2 Fig The performance of CNN + LSTM under different convolutional layers and kernel sizes. (JPG)
