# Supplementary material for: Deep6mA: A deep learning framework for exploring similar patterns in DNA N6-methyladenine sites across different species
Source: PLoS Comput Biol. 2021 Feb 18;17(2):e1008767. doi: 10.1371/journal.pcbi.1008767 (PMC7924747; doi:10.1371/journal.pcbi.1008767)
Supplement: S1 Table — (DOCX) [file pcbi.1008767.s001.docx]

**Table S1. The performance of CNN and CNN + LSTM based on 6mA-rice-Chen dataset under different CNN layers and kernel sizes.**

|  | | **Model** | **CNN**  **layers** | **Kernel size** | **SP**  **（%）** | **SN**  **（%）** | **ACC**  **（%）** | **MCC** | **AUC** |  |  |  |
| --- | --- | --- | --- | --- | --- | --- | --- | --- | --- | --- | --- | --- |
| CNN | | 1-256-5 | 1 | 5 | 75.34 | 58.82 | 67.08 | 0.35 | 0.72 |  |  |  |
|  |  | 1-256-8 | 1 | 8 | 90.91 | 45.64 | 68.27 | 0.41 | 0.80 |  |  |  |
|  |  | 1-256-10 | 1 | 10 | 91.14 | 48.07 | 69.60 | 0.44 | 0.81 |  |  |  |
|  |  | 1-256-16 | 1 | 16 | 88.36 | 69.50 | 78.93 | 0.59 | 0.86 |  |  |  |
|  |  | 2-256-5 | 2 | 5 | 88.43 | 64.61 | 76.52 | 0.55 | 0.85 |  |  |  |
|  |  | 2-256-8 | 2 | 8 | 80.09 | 80.61 | 80.35 | 0.61 | 0.88 |  |  |  |
|  |  | 2-256-10 | 2 | 10 | 83.57 | 85.50 | 84.53 | 0.69 | 0.92 |  |  |  |
|  |  | 2-256-16 | 2 | 16 | 95.00 | 78.89 | 86.94 | 0.75 | 0.94 |  |  |  |
|  |  | 3-256-5 | 3 | 5 | 76.57 | 80.66 | 78.61 | 0.58 | 0.86 |  |  |  |
|  |  | 3-256-8 | 3 | 8 | 85.39 | 85.05 | 85.22 | 0.71 | 0.92 |  |  |  |
|  |  | 3-256-10 | 3 | 10 | 90.11 | 85.43 | 87.77 | 0.76 | 0.94 |  |  |  |
|  |  | 3-256-16 | 3 | 16 | 91.07 | 85.41 | 88.24 | 0.77 | 0.95 |  |  |  |
|  |  | | | | | | | | |  |  |  |
| CNN  +LSTM | | 1-256-5-32 | 1 | 5 | 82.00 | 71.75 | 76.88 | 0.54 | 0.84 |  |  |  |
|  |  | 1-256-8-32 | 1 | 8 | 94.02 | 80.18 | 87.10 | 0.75 | 0.94 |  |  |  |
|  |  | 1-256-10-32 | 1 | 10 | 95.07 | 80.18 | 87.63 | 0.76 | 0.95 |  |  |  |
|  |  | 1-256-16-32 | 1 | 16 | 95.16 | 79.82 | 87.49 | 0.76 | 0.95 |  |  |  |
|  |  | 2-256-5-32 | 2 | 5 | 93.05 | 74.45 | 83.75 | 0.69 | 0.90 |  |  |  |
|  |  | 2-256-8-32 | 2 | 8 | 96.02 | 78.59 | 87.31 | 0.76 | 0.95 |  |  |  |
|  |  | 2-256-10-32 | 2 | 10 | 95.77 | 78.27 | 87.02 | 0.75 | 0.95 |  |  |  |
|  |  | 2-256-16-32 | 2 | 16 | 95.36 | 80.20 | 87.78 | 0.76 | 0.95 |  |  |  |
|  |  | 3-256-5-32 | 3 | 5 | 93.73 | 75.68 | 84.70 | 0.71 | 0.92 |  |  |  |
|  |  | 3-256-8-32 | 3 | 8 | 95.93 | 76.36 | 86.15 | 0.74 | 0.93 |  |  |  |
|  |  | 3-256-10-32 | 3 | 10 | 96.73 | 77.73 | 87.23 | 0.76 | 0.95 |  |  |  |
|  |  | 3-256-16-32 | 3 | 16 | 96.20 | 80.09 | 88.15 | 0.77 | 0.96 |  |  |  |
