# Supplementary material for: Deep6mA: A deep learning framework for exploring similar patterns in DNA N6-methyladenine sites across different species
Source: PLoS Comput Biol. 2021 Feb 18;17(2):e1008767. doi: 10.1371/journal.pcbi.1008767 (PMC7924747; doi:10.1371/journal.pcbi.1008767)
Supplement: S2 Table — (DOCX) [file pcbi.1008767.s002.docx]

**Table S2. The performance of CNN and CNN + LSTM based on *F.vesca* dataset under different CNN layers and kernel sizes.**

|  | | **Model** | **CNN**  **layers** | **Kernel size** | **SP**  **（%）** | **SN**  **（%）** | **ACC**  **（%）** | **MCC** | **AUC** |  |  |  |
| --- | --- | --- | --- | --- | --- | --- | --- | --- | --- | --- | --- | --- |
| CNN | | 1-256-5 | 1 | 5 | 71.15 | 72.30 | 71.73 | 0.44 | 0.79 |  |  |  |
|  |  | 1-256-8 | 1 | 8 | 88.13 | 65.03 | 76.56 | 0.55 | 0.87 |  |  |  |
|  |  | 1-256-10 | 1 | 10 | 87.55 | 68.80 | 78.16 | 0.58 | 0.88 |  |  |  |
|  |  | 1-256-16 | 1 | 16 | 83.93 | 86.91 | 85.42 | 0.71 | 0.93 |  |  |  |
|  |  | 2-256-5 | 2 | 5 | 85.13 | 84.17 | 84.65 | 0.69 | 0.92 |  |  |  |
|  |  | 2-256-8 | 2 | 8 | 75.15 | 94.34 | 84.76 | 0.71 | 0.94 |  |  |  |
|  |  | 2-256-10 | 2 | 10 | 82.47 | 97.07 | 89.78 | 0.80 | 0.97 |  |  |  |
|  |  | 2-256-16 | 2 | 16 | 93.59 | 94.70 | 94.15 | 0.88 | 0.98 |  |  |  |
|  |  | 3-256-5 | 3 | 5 | 70.26 | 94.30 | 82.30 | 0.67 | 0.93 |  |  |  |
|  |  | 3-256-8 | 3 | 8 | 84.17 | 97.06 | 90.62 | 0.82 | 0.98 |  |  |  |
|  |  | 3-256-10 | 3 | 10 | 88.45 | 97.11 | 92.78 | 0.86 | 0.98 |  |  |  |
|  |  | 3-256-16 | 3 | 16 | 88.83 | 96.98 | 92.91 | 0.86 | 0.98 |  |  |  |
|  |  | | | | | | | | |  |  |  |
| CNN  +LSTM | | 1-256-5-32 | 1 | 5 | 77.96 | 88.19 | 83.08 | 0.67 | 0.91 |  |  |  |
|  |  | 1-256-8-32 | 1 | 8 | 92.35 | 94.85 | 93.60 | 0.87 | 0.98 |  |  |  |
|  |  | 1-256-10-32 | 1 | 10 | 93.84 | 95.10 | 94.47 | 0.89 | 0.98 |  |  |  |
|  |  | 1-256-16-32 | 1 | 16 | 94.05 | 94.79 | 94.42 | 0.89 | 0.98 |  |  |  |
|  |  | 2-256-5-32 | 2 | 5 | 90.55 | 91.98 | 91.27 | 0.83 | 0.96 |  |  |  |
|  |  | 2-256-8-32 | 2 | 8 | 94.84 | 94.41 | 94.63 | 0.89 | 0.98 |  |  |  |
|  |  | 2-256-10-32 | 2 | 10 | 94.74 | 94.49 | 94.62 | 0.89 | 0.98 |  |  |  |
|  |  | 2-256-16-32 | 2 | 16 | 94.31 | 94.77 | 94.54 | 0.89 | 0.98 |  |  |  |
|  |  | 3-256-5-32 | 3 | 5 | 92.20 | 92.90 | 92.55 | 0.85 | 0.97 |  |  |  |
|  |  | 3-256-8-32 | 3 | 8 | 94.76 | 93.43 | 94.09 | 0.88 | 0.98 |  |  |  |
|  |  | 3-256-10-32 | 3 | 10 | 95.52 | 93.75 | 94.63 | 0.89 | 0.98 |  |  |  |
|  |  | 3-256-16-32 | 3 | 16 | 95.07 | 94.33 | 94.70 | 0.89 | 0.98 |  |  |  |
