# Supplementary figures and images for: Deep6mA: A deep learning framework for exploring similar patterns in DNA N6-methyladenine sites across different species
Source: PLoS Comput Biol. 2021 Feb 18;17(2):e1008767. doi: 10.1371/journal.pcbi.1008767 (PMC7924747; doi:10.1371/journal.pcbi.1008767)

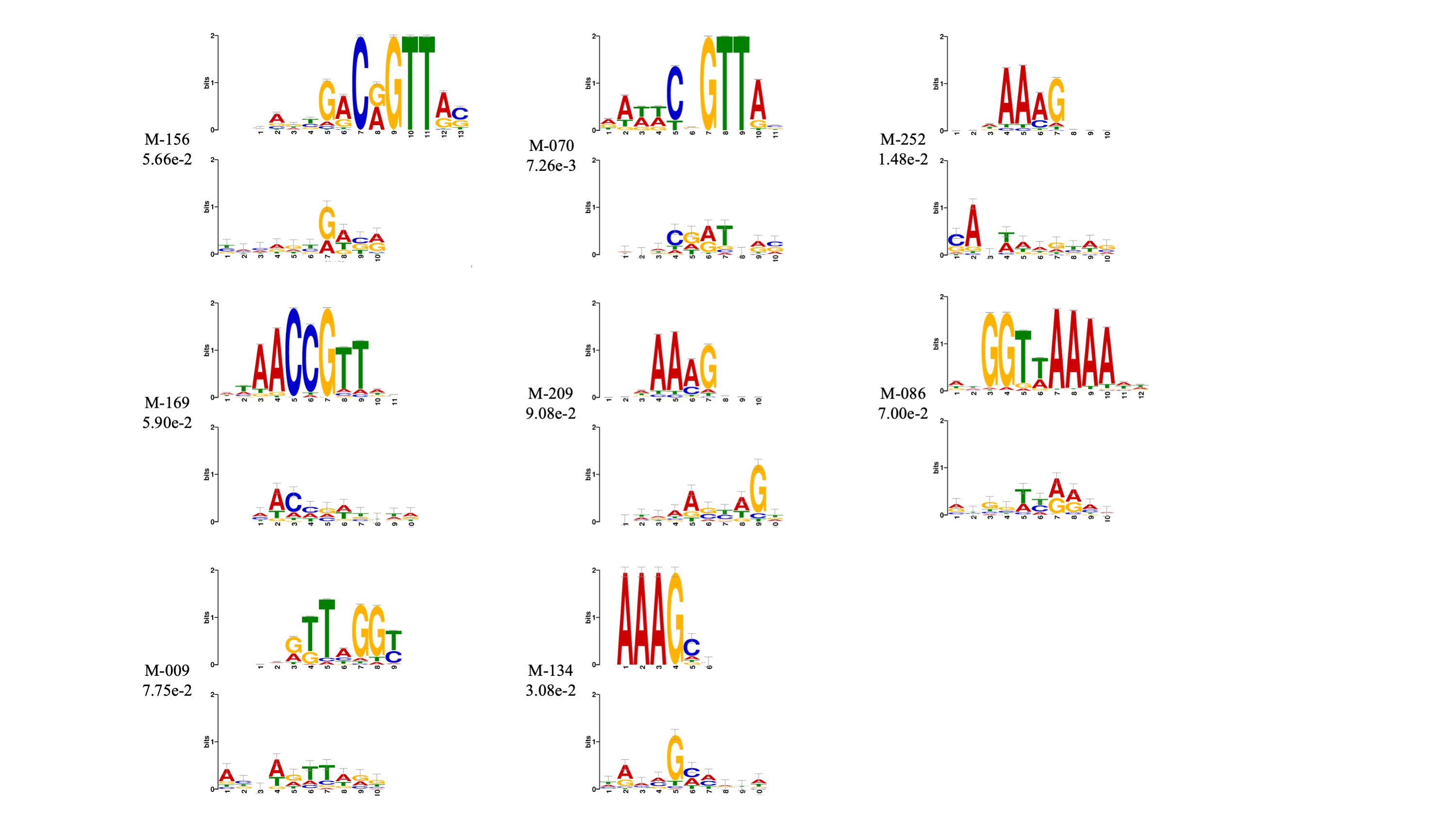

Supplement: S1 Fig — (JPG) [file pcbi.1008767.s004.jpg]

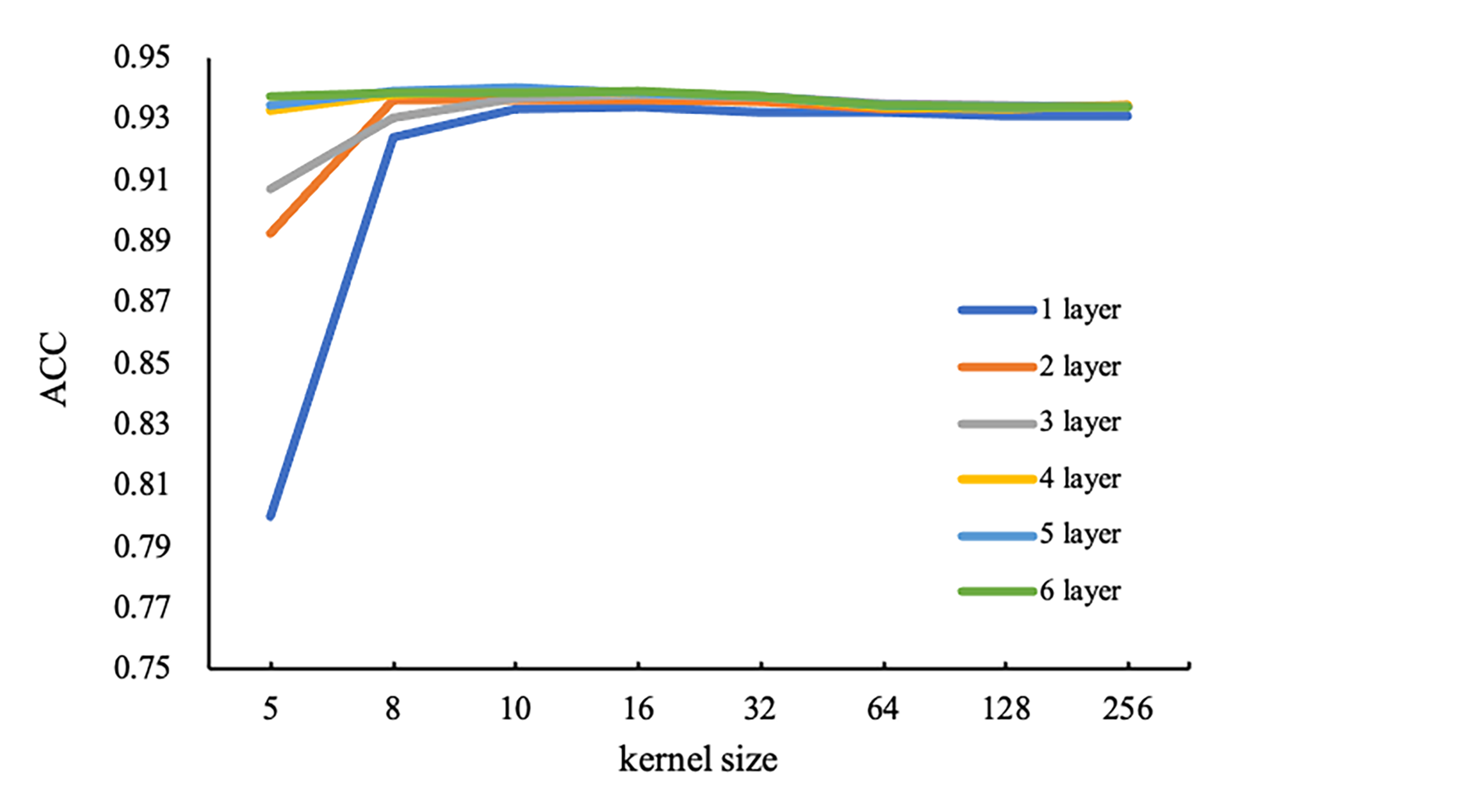

Supplement: S2 Fig — (JPG) [file pcbi.1008767.s005.jpg]
